# Effect of Ezetimibe on Insulin Secretion in db/db Diabetic Mice

**DOI:** 10.1155/2012/420854

**Published:** 2012-10-17

**Authors:** Yong Zhong, Jun Wang, Ping Gu, Jiaqing Shao, Bin Lu, Shisen Jiang

**Affiliations:** ^1^Department of Cardiology, School of Medicine, Nanjing University, Jinling Hospital, 305 East Zhongshan Road, Jiangsu Province, Nanjing 210002, China; ^2^Department of Endocrinology, School of Medicine, Nanjing University, Jinling Hospital, 305 East Zhongshan Road, Jiangsu Province, Nanjing 210002, China

## Abstract

*Objective*. To investigate the effect of ezetimibe on the insulin secretion in db/db mice. *Methods*. The db/db diabetic mice aged 8 weeks were randomly assigned into 2 groups and intragastrically treated with ezetimibe or placebo for 6 weeks. The age matched db/m mice served as controls. At the end of experiment, glucose tolerance test was performed and then the pancreas was collected for immunohistochemistry. In addition, in vitro perfusion of pancreatic islets was employed for the detection of insulin secretion in the first phase. *Results*. In the ezetimibe group, the fasting blood glucose was markedly reduced, and the total cholesterol (TC) and low-density lipoprotein cholesterol (LDL-C) were significantly lowered when compared with those in the control group (*P* < 0.05). At 120 min after glucose tolerance test, the area under curve in the ezetimibe group was significantly smaller than that in the control group (*P* < 0.05), but the AUC_INS0−30_ was markedly higher. In vitro perfusion of pancreatic islets revealed the first phase insulin secretion was improved. In addition, the insulin expression in the pancreas in the ezetimibe group was significantly increased as compared to the control group. *Conclusion*. Ezetimibe can improve glucose tolerance, recover the first phase insulin secretion, and protect the function of **β** cells in mice.

## 1. Introduction

Type 2 diabetes patients usually develop abnormal lipid metabolism concomitantly, and blood glucose control alone often fails to diminish the complications in the major blood vessels such as coronary artery in these patients. Thus, control of blood lipid is required in the glucose lowering therapy. A meta-analysis published in JAMA showed, when compared with moderate dose statins, intensive high-dose statin treatment may increase the risk for new diabetes [1]. The CORALL study showed high-dose statin might influence the blood glucose control in type 2 diabetes patients with concomitant dyslipidemia [2]. Ezetimibe is the first agent of a novel class of selective cholesterol absorption inhibitors and can be applied alone or in combination with statins in the treatment of type 2 diabetes with concomitant hyperlipidemia. Ezetimibe cannot only reduce the blood lipid but improve the glucose metabolism in type 2 diabetes patients [3]. Animal experiments reveal ezetimibe can reduce the fasting insulin and improve the high-lipid induced impaired glucose tolerance (IGT) in diabetic rats with insulin resistance, but the specific mechanism is still unclear [4]. In the present study, the effect of ezetimibe on the insulin secretion was investigated in db/db mice to explore the protective effect of ezetimibe on pancreatic islets. 

## 2. Materials and Methods 

### 2.1. Animals and Reagents

A total of 40 male db/db mice aged 8 weeks (specific pathogen free) and weighing 35~48 g) were randomly assigned into 2 groups (*n* = 20 per group). These animals were treated with ezetimibe (MSP SINGAPORE COMPANY LLC) at 10 mg/kg or placebo (5% gum Arabic) for 6 weeks. In addition, 20 aged matched db/m mice without diabetes in the same litter served as controls. The animals were processed in accordance with the Guide for the Care and Use of Laboratory Animals. 

### 2.2. Detection of Biochemical Variables

The body weight and blood glucose were measure weekly. Before treatment and after 6-week treatment, the fasting blood glucose, fasting TC, LDL-C, TG, and fasting serum insulin (FSI) were determined. The plasma insulin was measured with a mouse ELISA kit for insulin (Morinaga, Yokohama, Japan). The insulin sensitivity index (ISI) was calculated as follows: ISI = ln 1/(FBS  ×  FSI). HbA1c percentage was determined using the fully automated, high-pressure liquid chromatography Tosoh HLC-723 G8 analyzer (Japan). Plasma no-esterified fatty acid (NEFA, FFA) was measured by enzyme-like immunosorbent assay (ELISA) kit (Westang, Shanghai, China).

### 2.3. Intraperitoneal Glucose Tolerance Test (IPGTT)

After 6-week treatment, 10 mice were randomly selected from each group and received intraperitoneal glucose tolerance test (IPGTT). The area under curve (AUC) insulin was used to evaluate the insulin secretion, and AUC_INS0−30_ was employed for the assessment of the first phase insulin secretion. The AUC_INS0−30_ was calculated as follows: AUC_INS0−30_ = (INS_30_ − INS_0_) × 15. 

### 2.4. Evaluation of Pancreatic Islets with In Vitro Perfusion of Pancreatic Islets

The remaining 10 mice in each group were anesthetized with phenobarbital. The opening of the common bile duct duodenal papilla was clamped and common bile duct puncture was done followed by injection of 1 mg/mL type IV collagenase (2 mL). Then, the pancreas was rapidly collected and placed in Hank's solution containing type IV collagenase for digestion for 40 min. After washing under shaking, the pancreatic islets were separated. The in vitro islet perfusion system developed by our department was employed for perfusion of pancreatic islets. The perfusate was collected and the insulin concentration was detected to delineate the curve of first phase insulin secretion. 

### 2.5. Immunohistochemistry for Insulin

Animals were anesthetized with sodium phenobarbital and the pancreas was collected, fixed in 4% paraformaldehyde for 4~6 h and embedded in paraffin. ABC method was employed for the detection of insulin. The *β* cells positive for insulin in the pancreas were dark brown. 

### 2.6. Measurement of Pancreas

Image-Pro Plus 5.0.1 was used to measure the area of pancreas and that of *β* cells, and the content of *β* cells was calculated as follows: content of *β* cells (mg) = area of *β* cells/area of pancreas × weight of pancreas. 

### 2.7. Statistical Analysis

Statistical analysis was done with SPSS version 11.0 and data were expressed as mean ± standard deviation. Means among groups were compared with one way analysis of variance, and those between two groups were compared with LSD test. Data before and after experiment were compared with paired test. A value of *P* < 0.05 was considered statistically significant. 

## 3. Results

### 3.1. Biochemical Variables

At baseline, there were no marked differences in the FBG, HbA1c, FSI, TC LDL-C, TG, and FFA between ezetimibe group and control group. The db/db mice aged 8 weeks had the FBG of >15 mmol/L. After treatment with ezetimibe, the development of hyperglycemia was alleviated, and the blood glucose and HbA1c at the end of treatment in the db/db mice was markedly lower than that in the untreated db/db mice (*P* < 0.05), although there is no difference between before and after ezetimibe treatment. After ezetimibe treatment for 6 weeks, the TC, LDL-C, TG, and FFA in the ezetimibe group were markedly reduced as compared to the untreated db/db mice (*P* < 0.05). The ISI at baseline was comparable between ezetimibe-treated mice and untreated db/db mice, but the ISI in the ezetimibe-treated mice was significantly higher than that in the untreated db/db mice at the end of treatment (*P* < 0.05) (Table 1).

### 3.2. Effect of Ezetimibe on IPGTT in db/db Mice

**Figure 1:**
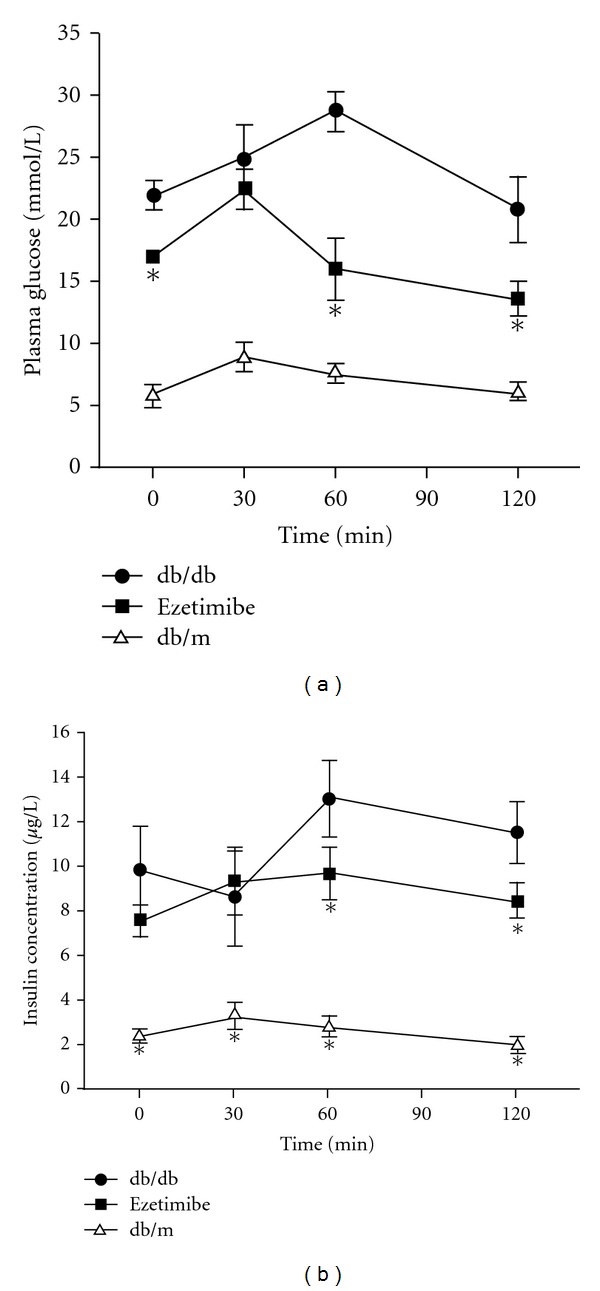
Effect of on IPGTT in db/db mice with diabetes. Compared with db/db, ezetimibe provided an improvement of glucose tolerance and first-phase insulin response. *P < 0.05.

At 120 min after glucose tolerance test, the blood glucose in the ezetimibe group was markedly reduced (*P* < 0.05) (Figure 1), and the AUC in the ezetimibe group was significantly lower than that in the untreated db/db mice (*P* < 0.05) (Figure 1). At 30 min after test, the plasma insulin increased and the AUC_INS0−30_ in the ezetimibe-treated mice was remarkably higher than that in the untreated db/db mice. This suggests that ezetimibe can improve the glucose tolerance and first phase insulin secretion (Figure 1). 

### 3.3. Effect of Ezetimibe on First Phase Insulin Secretion in db/db Mice

**Figure 2:**
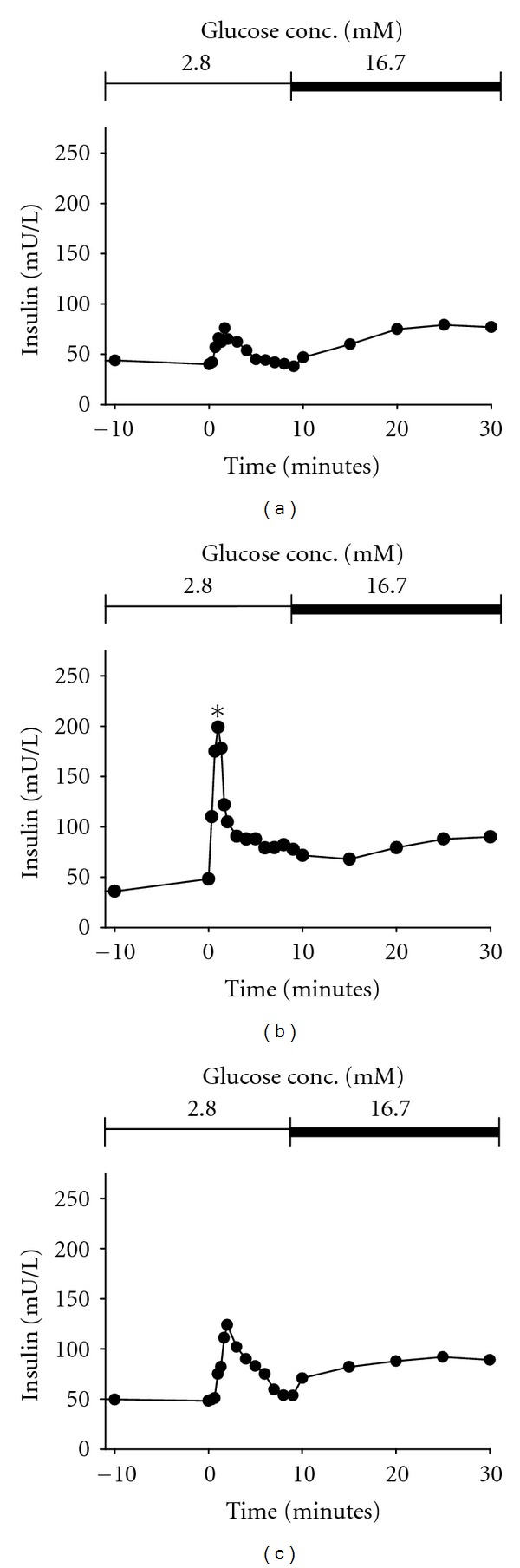
In vitro perfusion of pancreatic islets in different groups. (a), db/db; (b), db/m; (c), ezetimibe. Ezetimibe improved first-phase insulin response when compared with db/db, but the insulin response was still lower than db/m.

When compared with untreated db/db mice, the insulin secretion was not significantly increased in the ezetimibe-treated mice in the perfusion of pancreatic islets with low glucose solution, but the insulin secretion was markedly elevated at 1 min after perfusion with 16.7 mM glucose solution. This suggests that ezetimibe can improve the first phase insulin secretion, which, however, was still lower than that in the db/m group (Figure 2). 

### 3.4. Effect of Ezetimibe on Content of *β* Cells in db/db Mice

**Figure 3:**
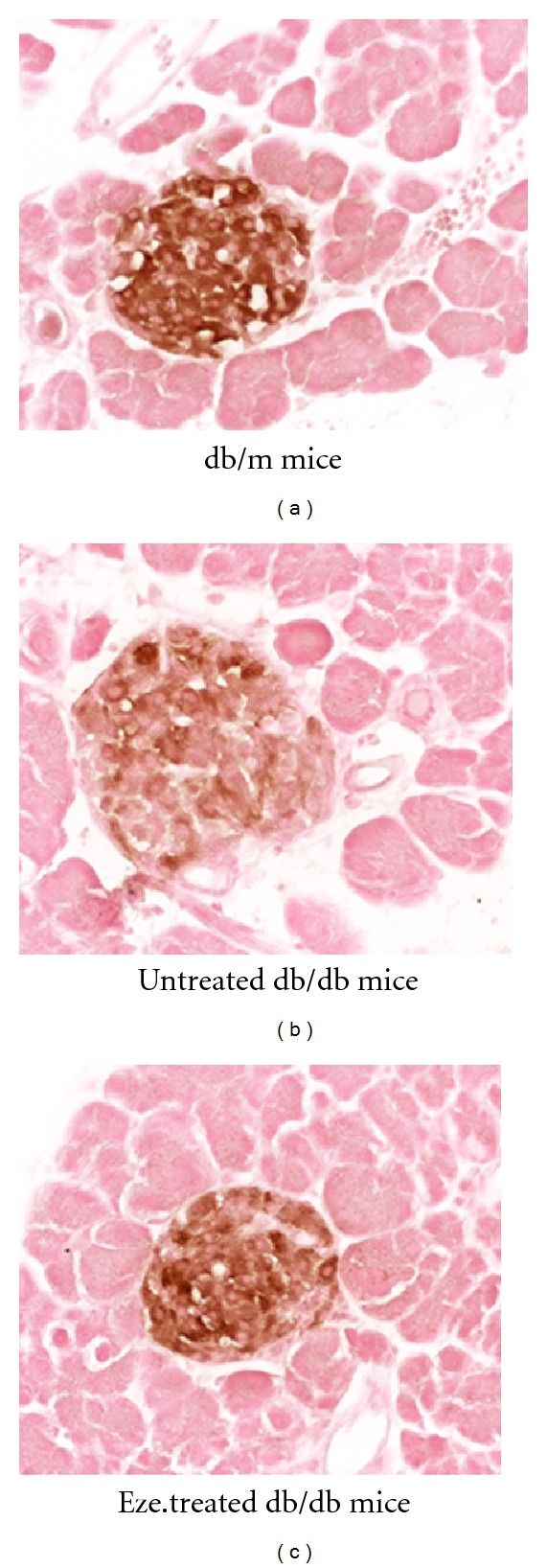
Immunohistochemistry for insulin in the pancreatic islets of three groups. Representative immunostaining for insulin performed with pancreatic tissue sections derived from db/m mice, placebo-treated db/db mice, and ezetimibe-treated db/db mice.

**Figure 4:**
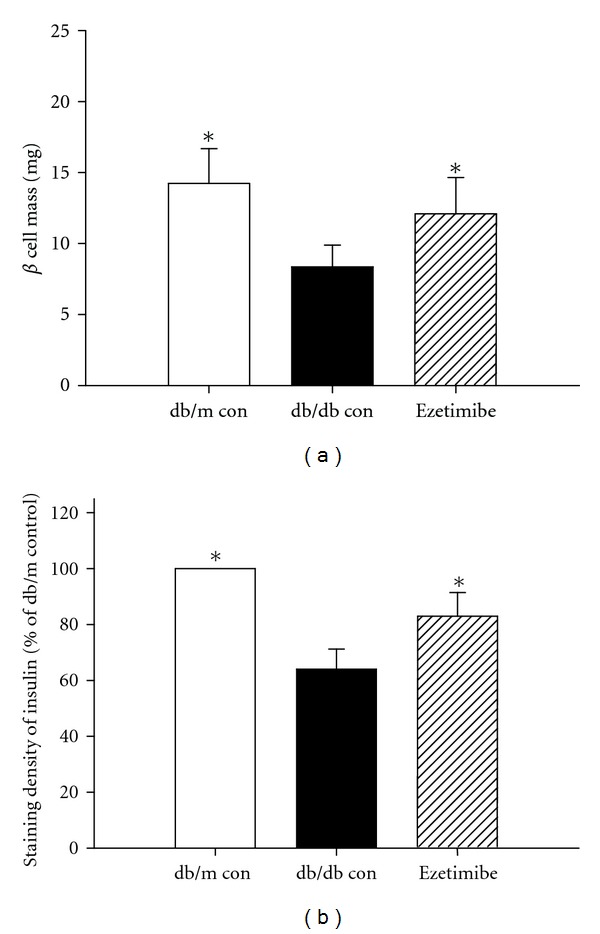
Content of *β* cells and staining intensity in three groups. (a) *β*-Cell mass was calculated by the following formula: islet *β*-cell mass (mg) = (the area stained by insulin antibody)/(the area of the whole pancreatic)∗(pancreas weight). (b) Insulin expression level was analyzed semiquantitatively. *P < 0.05.

After ezetimibe treatment for 6 weeks, quantitative analysis showed the content of *β* cells in the pancreatic islet was at a very low level in the db/db mice, but ezetimibe treatment could significantly reduce the *β* cell loss (Figures 3 and 4). Insulin staining intensity was determined by using the Scion Image Beta 4.0.3 for Windows (Maryland, USA). After ezetimibe treatment for 6 weeks, the *β*-cell staining intensity in the ezetimibe group was significantly higher than that in db/db group (Table 2).

## 4. Discussion

Ezetimibe is the first agent of a novel class of selective cholesterol absorption inhibitors and acts on the brush borders of rat small intestinal mucosal cells. Ezetimibe can inhibit the Niemann-Pick C1 Like 1 Protein (NPC1Ll) activity and selectively suppress the transportation of cholesterol in the diet and bile across the small intestine into the liver. Thus, the cholesterol stored in the liver is reduced which leads to reduction of synthesis of LDL receptor in the liver, promotion of LDL metabolism, and decrease in plasma LDL-C [5]. 

To date, studies have confirmed the NPC1Ll expression in not only the small intestine but the liver, pancreas, gallbladder, testis, and stomach [6]. Chan et al. found that, in patients with nonalcoholic fatty liver disease, ezetimibe could not only reduce the intrahepatic triglyceride and plasma levels of hsCRP, IL-6 and RBP-4 but also increase the adiponectin, which attributed to the improvement of hepatic steatosis, hepatic inflammation, and dyslipidemia and increase in the insulin sensitivity [7]. Nomura et al. [8] found that ezetimibe could inhibit the hepatic NPC1Ll activity, reduce the production of reactive oxygen species, suppress the JNK activity, reduce the unfolded or misfolded protein induced endoplasmic reticulum stress, promote the phosphorylation of PI-3K/AKT in the insulin signaling pathway, and then improve the insulin resistance in the liver. Although numerous studies have been conducted to investigate the hepatic insulin resistance, few studies report the effect of ezetimibe on the pancreatic islets. In the present study, we for the first time showed that ezetimibe-treated db/db mice not only reduce the blood lipids, but also alleviate blood high glucose, improve the glucose tolerance and first phase insulin secretion, elevate the insulin sensitivity, reduce the *β* cell loss, and protect the function of *β* cells. 

 Although there was no significant difference in the blood glucose level of db/db diabetic mice before and after ezetimibe treatment, the fasting glucose level, glucose tolerance, and glycosylated hemoglobin were all significantly lowered as compared with the control group. The abnormality of first phase insulin secretion is an early manifestation of dysfunction of *β* cells. Currently, methods aiming to evaluate the insulin secretion are used to detect the total insulin, which cannot reflect the changes in insulin in two phases and those in the amount of insulin. In the present study, we for the first time applied in vitro perfusion of pancreatic islets [9] to evaluate the changes in the first phase insulin secretion in db/db mice. Results revealed, in the ezetimibe group, the insulin secretion remained unchanged following perfusion with low glucose solution. However, at 1 min after perfusion with 16.7 mM glucose solution, the insulin secretion was markedly increased. Thus, we speculate that the improvement of hyperlipidemia in ezetimibe-treated mice might be attributed to the improvement of first phase insulin secretion. Naples et al. also found that ezetimibe treatment improved glucose tolerance, decreased fasting insulin levels in FFC-fed hamsters [10]. Hiramitsu et al. also found ezetimibe therapy reduced the fasting serum insulin level and HbA1c [11]. Although in SANDS study, there was an increase in blood glucose values in diabetes patients the treatment with statin and ezetimibe after 36 months of followup, the possible reason is probably related to the development of diabetes itself and the use of stains [12].

In our study, after ezetimibe treatment for 6 weeks, quantitative analysis showed ezetimibe could significantly reduce the *β* cell loss and increase the *β*-cell staining intensity. Consistent with our results, Yang et al. [13] found that, in diabetes mice with hyperlipidemia, long-term treatment with ezetimibe (20 weeks) could increase the number of *β* cells and the content of cytoplasmic insulin. 

The mechanism underlying the ezetimibe induced improvement of glucose metabolism under diabetic status is still poorly understood. Studies show that this might be related to the improvement of peripheral insulin resistance and regulation of signaling pathways. The persistent increase in plasma free fat acid (FFA) may elicit the insulin resistance in the muscles and liver, elevate the gluconeogenesis, and reduce the glucose uptake in the muscles. On the contrary, persistent reduction in FFA may improve the glucose tolerance and elevate the peripheral insulin sensitivity [14]. Ezetimibe can directly inhibit the cholesterol absorption, reduce the FFA, improve the insulin resistance, and reduce blood glucose. In addition, ezetimibe may promote the phosphorylation of PI-3K/AKT via suppressing the hepatic NPC1Ll activity and inhibit the JNK activity [8], which then improves the insulin resistance. However, the effect of ezetimibe on the insulin signaling pathway in the pancreatic islets is required to be further studied. 

Of course, there may be other mechanisms related to the ezetimibe improvement of insulin secretion. The incretin hormones, glucose-dependent insulinotropic polypeptide (GIP), and glucagon-like peptide-1 (GLP-1) are produced by the intestine and are released into the circulation in response to ingestion of macronutrients. Yang et al. had found that ezetimibe significantly active glucagon-like peptide-1  [13]. But Kikuchi et al. [15] had reported that the active glucagon-like peptide-1 (GLP-1) was not significantly affected by ezetimibe treatment in obese men. The inconsistent results and the mechanisms need to be further studied. 

Taken together, our results demonstrate that ezetimibe not only can reduce the serum lipids, but also can improve the first phase insulin secretion in *β* cells, alleviate blood high glucose, increase the insulin sensitivity, and protect the function of *β* cells in mice. Our findings provide a new strategy for the treatment of diabetes with concomitant hyperlipidemia and atherosclerosis.

## Figures and Tables

**Table:** (a)

Group	*n*	Body weight (g)	FBG (mmol/L)	FSI (ug/L)	TC (mmol/L)	LDL-C (mmol/L)	ISI
db/m	Baseline 20	29.10 ± 0.33*	5.51 ± 0.53*	2.43 ± 0.23*	2.65 ± 0.41*	1.65 ± 0.22*	−2.58 ± 0.15
	Terminal 20	35.80 ± 0.54*	5.71 ± 0.63*	2.63 ± 0.31*	2.55 ± 0.53*	1.79 ± 0.27*	−2.69 ± 0.19

db/db	Baseline 20	41.20 ± 3.18	17.30 ± 3.99	8.51 ± 1.26	5.14 ± 0.80	3.88 ± 0.44	−4.95 ± 0.32
	Terminal 20	50.20 ± 4.32	25.9 ± 2.00	9.55 ± 1.20	5.86 ± 0.85	4.91 ± 0.37	−5.49 ± 0.20

Ezetimibe	Baseline 20	40.92 ± 2.84	18.37 ± 4.25	9.01 ± 1.37	5.30 ± 0.77	3.69 ± 0.74	−5.07 ± 0.34
	Terminal 20	45.40 ± 3.81*	18.82 ± 3.99*	7.58 ± 0.67*	4.21 ± 0.63*	2.78 ± 0.62*	−4.38 ± 0.24

Note: db/m: db/m mice without diabetes; db/db: untreated db/db mice with diabetes; ezetimibe: ezetimibe-treated db/db mice with diabetes; *P < 0.05 untreated db/db mice versus treated db/db control mice.

**Table:** (b)

Group	*n*	TG (mmol/L)	FFA (mmol/L)	HbA1c (%)
db/m	Baseline 20	1.03 ± 0.22*	2.52 ± 0.39*	5.18 ± 1.21*
	Terminal 20	0.93 ± 0.27*	2.58 ± 0.48*	4.99 ± 1.03*

db/db	Baseline 20	1.99 ± 0.74	3.23 ± 0.87	8.80 ± 2.54
	Terminal 20	2.28 ± 0.49	3.74 ± 0.89	11.46 ± 2.87

Ezetimibe	Baseline 20	2.18 ± 0.45	3.35 ± 0.84	9.08 ± 2.67
	Terminal 20	1.50 ± 0.74*	2.88 ± 0.53*	9.77 ± 2.60*

Note: db/m: db/m mice without diabetes; db/db: untreated db/db mice with diabetes; ezetimibe: ezetimibe-treated db/db mice with diabetes; *P < 0.05 untreated db/db mice versus treated db/db control mice.

**Table 2:** Comparison of *β* cell amount and stain intensity among three groups.

Group	*β* cell amount (mg)	*β* cell stain intensity (%)
db/m control (*n* = 10)	14.23 ± 2.45*	100*
db/db control (*n* = 10)	8.36 ± 1.53	64.21 ± 7.21
Ezetimibe group (*n* = 10)	12.10 ± 2.55*	83.36 ± 8.45*

Versus db/db control mice, *P < 0.05.
